# Is Skin Coloration Measured by Reflectance Spectroscopy Related to Intake of Nutrient-Dense Foods? A Cross-Sectional Evaluation in Australian Young Adults

**DOI:** 10.3390/nu10010011

**Published:** 2017-12-23

**Authors:** Lee M. Ashton, Kristine B. Pezdirc, Melinda J. Hutchesson, Megan E. Rollo, Clare E. Collins

**Affiliations:** 1 School of Health Sciences, Faculty of Health and Medicine, University of Newcastle, Callaghan 2308, Australia; lee.ashton@newcastle.edu.au (L.M.A.); Kristine.pezdirc@newcastle.edu.au (K.B.P.); Melinda.hutchesson@newcastle.edu.au (M.J.H.); Megan.rollo@newcastle.edu.au (M.E.R.); 2 Priority Research Centre in Physical Activity and Nutrition, University of Newcastle, Callaghan 2308, Australia

**Keywords:** cross-sectional study, carotenoids, skin color, diet quality

## Abstract

The current study examines associations between the dietary intakes of nutrient-dense foods, measured using brief indices and skin coloration, measured using reflectance spectroscopy in young adults. This is a cross-sectional analysis of 148 young Australian males and females (55% female) aged 18 to 25 years. Dietary intake was assessed using a validated food frequency questionnaire, with responses used to calculate two dietary indices: (i) the Australian Recommended Food Score (ARFS); and (ii) the Fruit And Vegetable VAriety Score (FAVVA). Skin yellowness was measured at three body locations using reflectance spectroscopy. Associations were assessed using Spearman’s correlation coefficients, regression analysis, and agreement using weighted kappa (K_w_). Significant, moderate correlations were found between skin yellowness and diet index scores for the ARFS (ρ = 0.30, *p* < 0.001) and FAVVA score (ρ = 0.39, *p* < 0.001). These remained significant after adjustment for confounders (total fat intake, sex, skin lightness) and for agreement based on categorical rankings. Results suggest that measurement of skin coloration by reflectance spectroscopy can be used as an indicator of overall dietary quality and variety in young adults. Further exploration in diverse populations is required.

## 1. Introduction

Carotenoids are fat-soluble, yellow, orange, and red pigments found primarily in fruit and vegetables [1]. Small amounts of dietary carotenoids are also found in animal food sources, including fish, eggs and dairy products [1]. Carotenoids have been classified as antioxidants due to their ability to neutralize free radicals [2]. Human skin is directly exposed to ultraviolet (UV) radiation, tobacco smoke and ozone, which contribute to the production of free radicals [2]. Carotenoids accumulate in all layers of the skin, where they serve a protective role through neutralizing free radicals via the protective antioxidant chain in tissues [2].

Dietary sources of carotenoids are absorbed via the intestinal epithelial cells and enter the blood stream to be delivered to target tissues and organs, including all layers of human skin in particular the stratum corneum [3,4]. Carotenoids can be assayed using biochemical methods in blood samples or by non-invasive optical methods in human skin, such as reflectance spectroscopy or resonance raman spectroscopy to quantify the carotenoids present [5]. Both of these methods have been validated against plasma carotenoid concentrations [4,6,7]. Resonance raman spectroscopy detects skin carotenoids using a laser spectroscopy which probes the vibrational energy levels of a molecule [5]. Several studies have found positive correlations between diet (in particular fruit and vegetables) and skin carotenoids using this method [6,8,9]. Reflectance spectroscopy measures skin color using Commission Internationale de l’Eclairage (CIE) L*a*b* color space (where L* represents skin lightness and positive values of a* and b* represent degrees of redness and yellowness, respectively) [3]. The accumulation of dietary carotenoids in the skin contributes to the appearance of skin yellowness (b*) specifically [3]. Several studies have shown positive associations between skin yellowness (b*) and fruit and vegetable intake using reflectance spectroscopy [10,11,12]. A recent Randomized Controlled Trial (RCT) reported significant correlations between skin yellowness (b*), plasma carotenoid concentrations and the reported intake of high-carotenoid fruit and vegetables over a four-week period [13]. The findings from this study suggested that reflectance spectroscopy can be utilized as a quick non-invasive method for measuring dietary carotenoid intake and/or identifying low fruit and vegetable intake [13].

Diet quality scoring indices are commonly used as a method to identify both healthful and unhealthy dietary patterns. They have been designed to compare the nutritional adequacy of an individual’s dietary intake and how closely it aligns with the current guidelines [14]. The relationship between diet quality indices and nutrition-related health outcomes has been reviewed, which indicates that diet quality can predict biomarkers of disease and the risk of health outcomes including cardiovascular disease, some cancers, and all-cause and disease-specific mortality [14]. The validity of using dietary indices has been compared with objective measures, such as plasma carotenoids [15]. Plasma carotenoids are a biological marker of recent fruit and vegetable intake [16]. Plasma carotenoid concentrations have been shown to have positive correlations with sub-scale scores for fruit and vegetables within the Australian Recommend Food Score (AFRS) [15]. However, evaluation of plasma carotenoids as a biomarker of dietary intake is burdensome, expensive and invasive [15]. Reflectance spectroscopy offers an alternative objective measure of carotenoid intake that is non-invasive, rapid and less burdensome. However, this method has not been assessed or validated relative to brief dietary indices. Therefore, the aim of the current study was to evaluate the association between dietary intakes of nutrient dense foods, measured using brief indices and skin coloration, measured using reflectance spectroscopy in Australian adults aged 18 to 25 years.

## 2. Materials and Methods

### 2.1. Study Design

This secondary analysis uses cross-sectional data sets from two separate studies in young adults. Study one was a cross-sectional sample of 98 young men and women (*n* = 91, 93% female) [10], and study two was the baseline data from 50 young men who were recruited into a RCT targeting improved diet, physical activity and wellbeing [17]. The methods and primary analyses of both studies are published in detail elsewhere [10,17]. Study protocols for study one (H-2012-0217) and two (H-2015-0445) were approved by the University of Newcastle Human Research Ethics committee with the RCT also registered on the Australian New Zealand Clinical Trials Registry (ACTRN12616000350426). Written informed consent was obtained from all participants. Data from the two studies were combined in order to achieve a study population of young adults that was inclusive of both sexes. Data collection methods for the key outcome variables used in the current analysis were identical in both studies [10,17].

### 2.2. Participants

Complete participant demographics are published in detail elsewhere [10,17]. Briefly, the population samples in the current analysis were adults aged 18–25 years, recruited from the Hunter region of New South Wales, Australia and had completed both the Australian Eating Survey (AES) and reflectance spectroscopy measurement of skin coloration. Data were collected from October 2012 to June 2013 in study one and from March 2016 to May 2016 in study two. Eligibility criteria for study one were young adults that were non-smokers, and for study two were adults over 18 years old [10]. Key eligibility criteria for study two were being a young male (18–25 years), partaking in <300 min/week of combined moderate to vigorous physical activity, and consuming less than the age- and sex-specific national recommendations for fruit and vegetables intake [17]. However, as 97% of young Australian adults aged 18 to 24 years fail to meet national recommendations of two serves fruit/day and five-six serves of vegetable/day [18], only a very small proportion of men were excluded based on this.

### 2.3. Skin Coloration Measurement Using Reflectance Spectroscopy

The decision to use the reflectance spectroscopy method was due to its advantageous properties for skin carotenoid measurement including; its ability for quick and non-invasive assessments, high signal levels and its self-calibrating properties [5]. Despite previous concerns of reflectance spectroscopy having lower sensitivity in detecting carotenoids [5], recent research has found carotenoids characteristically absorb light in the 400–540 nm region of the spectrum and reflect back longer (yellow) wavelengths [10]. Therefore, wavelengths were set accordingly, to ensure greater specificity to various carotenoids and distinction from melanin and blood. In-person measurement of skin coloration was conducted using a handheld spectrophotometer at the University of Newcastle by researchers trained in standardized operating procedures for all measurements. KP undertook measurements in study one and trained LA, who undertook measurements in study two. Participants were advised prior to assessment not to wear make-up. All skin sites for reflectance spectroscopy assessment were cleaned using alcohol wipes with time allowed for the skin to dry. Skin coloration was measured using a hand-held CM700D spectrophotometer (Konica Minolta, Osaka, Japan) with an 8 mm diameter aperture, 2-degree observer angle and illuminant D65. The spectrophotometer was white point calibrated at each measurement session. Skin color (CIE L*a*b values) was recorded for each participant at three body locations on the left-hand side of the body unless stated otherwise. The three body locations included the inner arm (radiale), outer arm (medial humeral epicondyle) and palm (thenar muscle). The measurements were repeated three times at each site and the average recorded. Body locations were selected according to anatomical landmarks as specified in the ISAK International standards for anthropometric assessment [19]. Dietary carotenoids influence skin yellowness (b*) values and change in skin color [3]. As skin a* (redness) values are associated with skin blood perfusion [20] participants were asked to refrain from all physical activity for two hours prior to data collection. Since melanin affects both skin yellowness (b*) and lightness (L*) [21], this was adjusted for in the current analyses.

### 2.4. Dietary Indices

Data from the Australian Eating Survey food frequency questionnaire (AES FFQ) [22] were used to calculate two brief dietary indices: (i) The Australian Recommended Food Score (ARFS), previously shown to be a reliable and valid indicator of overall diet quality and variety [23]; and (ii) the Fruit And Vegetable VAriety Score (FAVVA), which assesses variety and frequency of fruit and vegetable intake. The AES FFQ is a self-administered 120 item semi-quantitative FFQ which assesses usual dietary intake over the past 6 months [22], previously shown to be valid and reliable in adults for assessing usual dietary intake [22].

**Australian Recommended Food Score (ARFS):** uses a sub-set of 70 questions from AES FFQ related to core nutrient-dense foods. The total ARFS score is calculated by summing points within eight sub-scales (Supplementary file 1), based on usual weekly intake of specific foods and beverages whose consumption aligns with the Australian Guide to Healthy Eating (AGHE) within the Australian Dietary Guidelines [24]. There are 20 questions related directly to vegetable intake, 12 to fruit, 13 to protein foods (seven to meat and six to vegetarian sources of protein), 12 to breads/cereals, 10 to dairy foods, one to water, and two to spreads/sauces. The total score ranges from zero to a maximum of 73 points. Briefly, most items in the AES FFQ frequency response options are collapsed into two categories ‘once per week or more’ or ‘less than once per week or never’. For most foods, respondents were awarded one point for a reported consumption of ‘once per week or more’, but differed for some items depending on national dietary guideline recommendations with consideration of the AGHE [24]. A higher total score is indicative of more optimal nutrient intakes, greater variety within the core food groups and alignment with Australian Dietary Guidelines [23,25]. Some of the food items for meat (i.e., beef, lamb) and dairy (i.e., ice-cream, frozen yoghurt) had a limit placed on their score for higher intakes, due to higher intakes being associated with potentially higher saturated fat or disease risk. Additional points were awarded for greater consumption of vegetables with evening meals, and healthier choices for bread and milk. Table S1 summarizes the detailed scoring method for items in the ARFS.

**Fruit and Vegetable VAriety Score:** uses a sub-set of 35 questions from AES FFQ related to usual intake frequency of a variety of vegetables and fruits across a comprehensive range of those consumed by the general Australian population. The FAVVA score was developed and modeled based on a previous fruit and vegetable index [26]. This total FAVVA score is calculated by summing points awarded from the fruit and vegetable sub-scales with 23 questions related directly to usual intake frequency of vegetables and legumes, and 12 questions about fruit intake. The FAVVA score uses all the fruit and vegetable questions from the AES FFQ except for the two vegetable questions relating to intake of ‘hot chips’. The total score ranges from 0 to a maximum of 190 points. Table S2 summarizes the detailed scoring method for items in the FAVVA. Briefly, for most items, 0 points were awarded for a report of ‘Never’ consumed, and then points were awarded incrementally for more frequent intake, with 1 point for ‘<once per month’, 2 points for ‘1–3 times per month’, 3 points for ‘once per week’, 4 points for ‘2–4 times per week’ and 5 points for those reporting ‘≥5 times per week’. A number of frequently consumed fruit (apples, bananas and oranges) and vegetable (peas, broccoli, carrots and lettuce) items had a scoring range to reflect more frequent consumption to account for this, with 6 points awarded for ‘once per day’ and 7 points for ‘2 or more times per day’.

Total energy intake (kJ/day) and total fat intake (g/day) was calculated from the AES FFQ. Total servings of fruit and vegetables/day were calculated by summing the weight of relevant food items estimated by the AES FFQ, divided by the standard serving size dictated by the Australian Guide to Healthy Eating (fruit serving 150 g, vegetable serving 75 g) [24]. Height was measured using a portable BSM370 stadiometer correct to 0.1 cm using the stretch stature method and weight was measured using the Inbody720 Body Composition Analyzer (Biospace Co., Ltd., Seoul, Korea). Body mass index (BMI) was calculated using the standard equation (weight kg/height m^2^). Age and sex were recorded by questionnaire.

### 2.5. Statistical Analysis

Data were analyzed using Stata Version 12 (StataCorp. 2011. Stata Statistical Software: StataCorp LP, College Station, TX, USA) using an alpha level of 0.05. The relationship between skin yellowness due to dietary carotenoid (overall b* value calculated as the average across the three body sites at the radiale, medial humeral epicondyle and palmar thenar muscle) and the two dietary indices (ARFS and FAVVA) were evaluated in three ways. Firstly, Spearman’s correlation coefficients, due to the non-normal distribution of dietary intake, were used to compare the strength of the linear relationship between overall b* and diet index scores. Correlation strength was described as poor <0.20, moderate 0.2–0.6, or strong >0.6, as previously identified within dietary validation studies [27,28]. Secondly, linear regression models were used to examine how much of the variation in skin yellowness (overall b* value) was explained by scores for each diet index. This included an unadjusted model and an adjusted model. The unadjusted model did not account for any confounders, while the adjusted model included the following potential confounders in the model; sex, skin lightness (L*)—as melanin affects both skin yellowness (b*) and lightness (L*) [21]—and total fat intake (g/day)—as carotenoids are fat soluble and bioavailability is affected by dietary fat [29]. The regression models were bootstrapped [30] to obtain estimates of the standard errors of the coefficients with replications in the order of 100 [31]. *R*^2^ values and regression coefficients (95% Confidence Intervals) are also reported. Lastly, the precision of agreement between categorical ranking of skin yellowness (b*) and diet index score by tertiles was tested using weighted Kappa (K_w_) statistics.

## 3. Results

### 3.1. Study Population

A total of 148 adults completed the AES FFQ and had their skin coloration measured. The key characteristics of these participants are summarized in Table 1.

### 3.2. Association between Skin Coloration Overall b* (Skin Yellowness) and Diet Indices

Table 2 summarizes the spearman’s correlations and linear regression analyses (unadjusted and adjusted) for all associations. Table 3 summarizes the extent of agreement between tertiles of skin yellowness (b*), and each dietary index and sub-scale variable using weighted Kappa statistics.

(i) Spearman’s Correlation

**ARFS:** There was a statistically significant moderate positive correlation between total ARFS and skin yellowness b* (ρ = 0.30, *p* < 0.001), and for the ARFS sub-scales of fruit (ρ = 0.30, *p* < 0.001), and vegetarian alternatives (ρ = 0.35, *p* < 0.001). Although the vegetable sub-scale was significantly correlated (*p* < 0.05) with skin yellowness b*, this association was classified as poor (ρ < 0.2). No other associations with sub-scales reached statistical significance.

**FAVVA:** For the total FAVVA score there were statistically significant, moderate positive correlations with skin yellowness b* (ρ = 0.39, *p* < 0.001), and the sub-scales of fruit (ρ = 0.37, *p* < 0.001) and vegetables (ρ = 0.30, *p* < 0.001) (Table 2).

(ii) Linear Regression Analysis

**ARFS:** In the unadjusted regression model, there were statistically significant positive associations between total ARFS and skin yellowness b* (β = 0.07, *p* < 0.001), and for the ARFS sub-scales of fruit (β = 0.20, *p* < 0.01) vegetables (β = 0.08, *p* < 0.05), and vegetarian alternatives (β = 0.60, *p* < 0.001). The significant associations between skin yellowness (b*) and total ARFS (β = 0.04, *p* < 0.05) and vegetarian alternatives (β = 0.38, *p* < 0.001) remained statistically significant in the fully adjusted regression model. In this fully adjusted model, the spreads/sauces sub-scale was negatively associated with skin yellowness b* (β = −0.50, *p* < 0.05).

**FAVVA:** In the unadjusted regression model, there were statistically significant positive associations between total FAVVA and skin yellowness b* (β = 0.03, *p* < 0.001), and for the FAVVA sub-scales of fruit (β = 0.08, *p* < 0.001) and vegetables (β = 0.04, *p* < 0.001), all of which remained significant in the fully adjusted regression analyses (Table 2).

(iii) Agreement Using Weighted Kappa (K_w_) Statistics

**ARFS:** Level of agreement based on categorical rankings indicated significant agreement by tertile of total ARFS and skin yellowness b* (K_w_ = 0.21, *p* < 0.001), and also for the sub-scales of vegetables (K_w_ = 0.14, *p* < 0.05), fruit (K_w_ = 0.20, *p* < 0.001), vegetarian alternatives (K_w_ = 0.23, *p* < 0.001) and grains (K_w_ = 0.16, *p* < 0.01) (Table 3).

**FAVVA:** Level of agreement based on categorical rankings was significant for total FAVVA score (K_w_ = 0.22, *p* < 0.001), FAVVA fruit sub-scale (K_w_ = 0.24, *p* < 0.001) and FAVVA vegetable sub-scale (K_w_ = 0.19, *p* < 0.01) when compared to tertiles of skin yellowness (b*) (Table 3).

## 4. Discussion

This is the first study to explore the associations between brief dietary indices reflecting intakes of nutrient-dense, healthy foods and skin coloration, measured objectively using skin reflectance spectroscopy. Results indicate that in young adults aged 18 to 25 years, a higher diet quality score and a high fruit and vegetable variety scores, as assessed using the ARFS and the FAVVA indices, was related to higher skin yellowness (b*). This was demonstrated through statistically significant positive correlations between dietary index scores and skin yellowness (b*), and also by agreement (Kappa) across quantiles, which remained significant in linear regression analyses adjusted for sex, total fat intake (g/day), and skin lightness (L*). The total ARFS score and sub-scales for fruit and vegetarian alternatives were moderately correlated with skin yellowness (b*). While the total FAVVA score and sub-scales for fruit and vegetables were also moderately correlated with skin yellowness (b*). This suggests that measurement of skin yellowness using reflectance spectroscopy could potentially be used as an efficient and objective way of predicting overall dietary quality and intakes of carotenoid-rich foods. However, the moderate correlations in the current study may not be considered large enough to have confidence in using the reflectance spectroscopy as a diagnostic tool and further research is required to explore these associations in larger and more diverse populations.

For ARFS, the association with vegetarian alternative foods was not expected, but may be due to the high carotenoid content of foods in this category, specifically lutein, zeaxanthin and β-carotene, found in legumes such as lentils, beans and chickpeas [32]. Previous research has consistently found higher intakes of foods containing dietary carotenoids are associated with higher skin yellowness (b*). For example a cross-sectional study in young men and women in the UK found significant positive correlations between skin yellowness and dietary β-carotene intake (ρ = 0.29, *p* = 0.013) after controlling for exercise [11]. A recent RCT in young Australian women that examined the impact of consuming foods high versus low in dietary carotenoids found significant positive correlations between the change in skin yellowness (b*) and change in plasma carotenoid concentrations of α-carotene (ρ = 0.29, *p* < 0.05), β-carotene (ρ = 0.35, *p* < 0.001) and total carotenoids (ρ = 0.27, *p* < 0.05) [13].

The negative relationship between ARFS spreads/sauces sub-scale with skin yellowness was not expected, and may have reduced the association with the total ARFS score. For each additional point scored for spreads/sauces sub-scale, there was a decrease in 0.50 units in overall skin yellowness. This sub-scale was included in the diet quality score because yeast extract spread and tomato ketchup/barbecue sauce contain a large amount of B-group vitamins and β-carotene (respectively) [33], which have been shown to be associated with skin color [11,13]. This suggested that higher scores in this population potentially reflect co-consumption of foods low in dietary carotenoids that may displace nutrient-dense foods. An example of this would be tomato sauce with a meat pie, but without vegetables. Further research into the dietary patterns in this specific age group is warranted. In addition, the fat-soluble nature of dietary carotenoids may help explain the negative relationship with spreads/sauces sub-scale [29].

Total FAVVA score and sub-scales for fruit and vegetables were all significantly associated with skin yellowness (b*). The FAVVA score reflects frequency and variety of fruit and vegetables consumed, and therefore these results were expected, given that previous studies have also reported higher fruit and vegetable consumption to be associated with higher skin yellowness (b*) [11,12,13]. The correlation coefficients in the current analysis are comparable to a cross-sectional study in 82 young men and women in the UK, which demonstrated a significant correlation (ρ = 0.25, *p* = 0.03) between usual fruit and vegetable intakes and skin yellowness after controlling for exercise [11]. Another study that examined change in fruit and vegetable intakes in 35 young men and women over a six-week period [12] found a modest increase in intake was associated with a significant increase in skin yellowness (*b* = 0.31, *p* = 0.05). These significant relationships highlight the sensitivity of reflectance spectroscopy in detecting carotenoids in skin, which is important, given young adults generally have low fruit and vegetables intakes [18]. In Australia; the most recent National Health Survey reported that 97% of young Australian adults aged 18 to 24 years fail to meet national recommendations of two serves fruit/day and 5–6 serves of vegetable/day [18]. Reported intakes of vegetables and fruit were higher in the current study and although some of the differences could be explained by the different dietary methods used to quantify intake, the majority of young adults still failed to achieve national recommendations (*n* = 124/148, 84%). An important insight from the current analysis is the potential for reflectance spectroscopy to predict diet intake of individuals across a range of differing intakes, and the ability to identify those who consume a greater variety of vegetables and fruit more frequently.

### 4.1. Implications for Research

Traditional methods of dietary assessment can be burdensome and resource intensive [34]. Results from the current study highlight that a rapid, non-invasive assessment of skin coloration, measured objectively using reflectance spectroscopy at three body sites may be sufficient to estimate overall diet quality and frequency and variety of usual dietary intake, particularly for fruit and vegetables. This is important for population groups where recall of dietary intake is challenging, or where resources mean that only a brief assessment of diet is warranted.

Diet quality has consistently been shown to have an inverse relationship with all-cause morbidity and mortality [14,35], while consuming a greater variety of fruit and vegetables is associated with lower odds of developing metabolic syndrome, obesity, hypercholesterolemia and hypertension [36,37]. As such, the associations between skin yellowness measured using reflectance spectroscopy and the dietary indices evaluated in the current study may therefore be indicative of disease risk. Future research should examine whether skin coloration, measured using reflectance spectroscopy, can predict health status and disease risk in diverse populations.

### 4.2. Strengths and Limitations

A key limitation of the current study is the cross-sectional design, and hence only associations between intake and skin yellowness could be evaluated. In addition, the diversity of the samples of dietary intakes across the two datasets due to differences in eligibility criteria may have affected the ability to identify stronger associations. In addition, responses from the AES FFQ were self-reported and therefore subject to reporting bias [38]. While other studies have explored relationships between carotenoids and skin redness [12,39], this was not assessed in the current study, due to the greater contribution of dietary carotenoids in the skin on the appearance of skin yellowness (b*) [3]. Strengths include use of an objective measure of skin color using reflectance spectroscopy with assessments taken at standardized anthropometric sites and operating procedure. In addition, to the best of the authors’ knowledge, this is the first evaluation of skin coloration in relation to brief dietary indices in a relatively large number of adults.

## 5. Conclusions

The present study indicates that overall diet quality and fruit and vegetable variety, assessed using two brief dietary indices (ARFS and FAVVA), were significantly associated with skin yellowness (b*), although those associations were weaker after adjustment for skin lightness (L*), sex, and total fat intake. This suggests that measurement of skin coloration using reflectance spectroscopy could potentially be used as a predictor of overall dietary quality and variety. Future research should examine these associations in more diverse populations and also examine whether skin coloration can predict health status and disease risk.

## Figures and Tables

**Table 1:** Characteristics of participants (*n* = 148).

Characteristic	Mean ± SD or *n* (%)
Age (years)	21.7 ± 2.2
Female	82 (55.4%)
Weight (kg)	70.9 ± 15.8
Height (cm)	171.7 ± 9.6
BMI (kg/m^2^)	23.9 ± 4.1
Energy intake (kJ/day)	9238.0 ± 3004.9
Fat intake (g/day)	78.4 ± 28.6
Fruit serves/day	1.8 ± 1.5
Vegetable serves/day	4.4 ± 2.4
ARFS (total possible score)	
Total Score (73)	32.5 ± 9.8
Vegetables (21)	11.7 ± 5.0
Fruit (12)	5.1 ± 3.2
Protein–Meat (7)	2.3 ± 1.4
Protein–Vegetarian sources (6)	2.0 ± 1.4
Breads/cereals–Grains (13)	5.5 ± 2.2
Dairy (11)	4.2 ± 1.8
Spreads/Sauces (2)	1.0 ± 0.8
FAVVA (total possible score)	
Total Score (190)	85.1 ± 25.4
Vegetables (122)	56.3 ± 17.2
Fruit (68)	28.8 ± 11.6
Skin coloration reflectance spectroscopy	
Overall L*	64.3 ± 3.6
Overall a*	8.6 ± 1.5
Overall b*	16.7 ± 2.4

L* values represent skin lightness, a* values represent skin redness and b* values represent skin yellowness. Overall values represent average skin color across the three body sites (radiale, medial humeral epicondyle and palmar thenar muscle). Abbreviations: SD = Standard Deviation, BMI = Body Mass Index, ARFS = Australian Recommended Food Score, FAVVA= Fruit And Vegetable VAriety Score.

**Table 2:** Spearman’s correlations and regression analyses (unadjusted and adjusted) between participant (*n* = 148) skin yellowness (overall b*) and diet index.

	Spearman’s ρ	Unadjusted Regression				Adjusted Regression			
		β	95% CI	*R*^2^	SE	β	95% CI	*R*^2^	SE
**ARFS**									
Total ARFS	0.30 ***	0.07 ***	0.03, 0.10	0.08	0.02	0.04 *	0.00, 0.07	0.34	0.02
ARFS–Vegetables	0.19 *	0.08 *	0.01, 0.15	0.03	0.04	0.05	−0.01, 0.11	0.33	0.03
ARFS–Fruit	0.30 ***	0.20 **	0.08, 0.32	0.07	0.06	0.09	−0.02, 0.20	0.33	0.06
ARFS Meat	0.14	0.28	−0.03, 0.59	0.03	0.16	0.20	−0.09, 0.50	0.33	0.15
ARFS Vegetarian alternatives	0.35 ***	0.60 ***	0.34, 0.85	0.13	0.13	0.38 **	0.11, 0.64	0.37	0.13
ARFS Grains	0.11	0.09	−0.06, 0.25	0.01	0.08	0.06	−0.08, 0.20	0.32	0.07
ARFS Dairy	0.08	0.10	−0.09, 0.28	0.01	0.10	−0.02	−0.19, 0.14	0.32	0.08
ARFS Spreads/sauces	−0.12	−0.44	−0.94, 0.07	0.02	0.26	−0.50 *	−0.92, −0.09	0.34	0.21
**FAVVA**									
Total FAVVA	0.39 ***	0.03 ***	0.02 0.05	0.14	0.01	0.02 ***	0.01, 0.04	0.38	0.01
FAVVA fruit	0.37 ***	0.08 ***	0.05, 0.10	0.14	0.01	0.05 ***	0.02 0.08	0.37	0.01
FAVVA veg	0.30 ***	0.04 ***	0.02, 0.06	0.09	0.01	0.03 **	0.01, 0.04	0.35	0.01

Adjusted regression models were adjusted for total fat intake, sex, skin lightness. β = Regression coefficient. CI = Confidence Interval, SE = Bootstrap standard error. *R*^2^ = Partial Correlation coefficient. ARFS = Australian Recommended Food Score, FAVVA = Fruit and Vegetable VAriety * *p*-value < 0.05; ** *p*-value < 0.01; *** *p*-value < 0.001.

**Table 3:** Extent of agreement measured using weighted Kappa (K_w_) statistics between tertiles of skin yellowness (overall b*) and two dietary indices: (i) The Australian recommended Food Score (ARFS) and (ii) the Fruit and Vegetable VAriety Score (FAVVA).

Variable	*n* = 148 (100%)			Kappa (K_w_)	*p*-Value
	Same Tertile	Adjacent Tertile	Misclassified ^a^		
ARFS					
Total ARFS	65 (44%)	62 (42%)	21 (14%)	0.21	<0.001
ARFS−Vegetables	59 (40%)	63 (43%)	26 (18%)	0.14	<0.05
ARFS−Fruit	63 (43%)	66 (45%)	19 (13%)	0.20	<0.001
ARFS Meat	52 (35%)	69 (47%)	27 (18%)	0.08	0.09
ARFS Vegetarian alternatives	68 (46%)	63 (43%)	17 (11%)	0.23	<0.001
ARFS Grains	63 (43%)	64 (43%)	21 (14%)	0.16	<0.01
ARFS Dairy	56 (38%)	57 (39%)	35 (24%)	0.09	0.08
ARFS Spreads/sauces	45 (30%)	49 (33%)	54 (36%)	-0.06	0.86
FAVVA					
Total FAVVA	60 (41%)	73 (49%)	15 (10%)	0.22	<0.001
FAVVA fruit	65 (44%)	66 (45%)	17 (11%)	0.24	<0.001
FAVVA veg	65 (44%)	60 (44%)	23 (16%)	0.19	<0.01

^a^ Misclassifed: classified as extreme categories.

## Supplementary Materials

The following are available online at http://www.mdpi.com/2072-6643/10/1/11/s1, Table S1: Scoring method for items in the ARFS, Table S2: Scoring method for items in the FAVVA index.
