# Supplementary material for: Is Skin Coloration Measured by Reflectance Spectroscopy Related to Intake of Nutrient-Dense Foods? A Cross-Sectional Evaluation in Australian Young Adults
Source: Nutrients. 2017 Dec 23;10(1):11. doi: 10.3390/nu10010011 (PMC5793239; doi:10.3390/nu10010011)
Supplement: Supplementary file 1 [file nutrients-10-00011-s001.zip › Supplementary Table 2_FAVVA.docx]

| **Supplementary Table 2:** Scoring method for items in the FAVVA index | |
| --- | --- |
| **1 Vegetables** | |
| **D. How many times a week do you eat vegetables with your meal at night? (not including hot chips)** |  |
| O Never O Less than once per week  O 1-2 per week  O 3-4 per week  O 5 or more per week | 0  1  2  3  4 |
| **F3. Potato boiled, mashed, baked (1 serving)** |  |
| O Never  O Less than 1 per month  O 1-3 per month  O Once per week O 2-4 per week O 5 or more per week | 0  1  2  3  4  5 |
| **F4. Pumpkin (1 serving)** |  |
| O Never  O Less than 1 per month  O 1-3 per month  O Once per week O 2-4 per week O 5 or more per week | 0  1  2  3  4  5 |
| **F5. Sweet potato (1 serving)** |  |
| O Never  O Less than 1 per month  O 1-3 per month  O Once per week O 2-4 per week O 5 or more per week | 0  1  2  3  4  5 |
| **F6. Cauliflower (1 serving)** |  |
| O Never  O Less than 1 per month  O 1-3 per month  O Once per week O 2-4 per week O 5 or more per week | 0  1  2  3  4  5 |
| **F7. Green beans (1 serving)** |  |
| O Never  O Less than 1 per month  O 1-3 per month  O Once per week O 2-4 per week O 5 or more per week | 0  1  2  3  4  5 |
| **F8. Spinach (1 serving)** |  |
| O Never  O Less than 1 per month  O 1-3 per month  O Once per week  O 2-4 per week O 5 or more per week | 0  1  2  3  4  5 |
| **F9. Cabbage or brussel sprouts (1 serving)** |  |
| O Never  O Less than 1 per month  O 1-3 per month  O Once per week O 2-4 per week O 5 or more per week | 0  1  2  3  4  5 |
| **F10. Peas (1 serving)** |  |
| O Never  O Less than 1 per month  O 1-3 times per month  O Once per week O 2-4 times per week O 5-6 times per week O Once per day O 2 or more times per day | 0  1  2  3  4  5  6  7 |
| **F11. Broccoli (1 serving)** |  |
| O Never  O Less than 1 per month  O 1-3 times per month  O Once per week O 2-4 times per week O 5-6 times per week O Once per day O 2 or more times per day | 0  1  2  3  4  5  6  7 |
| **F12. Carrots (1 serving)** |  |
| O Never  O Less than 1 per month  O 1-3 times per month  O Once per week O 2-4 times per week O 5-6 times per week O Once per day O 2 or more times per day | 0  1  2  3  4  5  6  7 |
| **F13. Zucchini, eggplant, squash (1 serving)** |  |
| O Never  O Less than 1 per month  O 1-3 per month  O Once per week O 2-4 per week O 5 or more per week | 0  1  2  3  4  5 |
| **F14. Capsicum (1 serving)** |  |
| O Never  O Less than 1 per month  O 1-3 per month  O Once per week O 2-4 per week O 5 or more per week | 0  1  2  3  4  5 |
| **F15. Corn, sweetcorn, corn on the cob (1 serving)** |  |
| O Never  O Less than 1 per month  O 1-3 per month  O Once per week  O 2-4 per week O 5 or more per week | 0  1  2  3  4  5 |
| **F16. Mushroom (1 serving)** |  |
| O Never  O Less than 1 per month  O 1-3 per month  O Once per week O 2-4 per week O 5 or more per week | 0  1  2  3  4  5 |
| **F17. Tomatoes (1 serving)** |  |
| O Never  O Less than 1 per month  O 1-3 per month  O Once per week O 2-4 per week O 5 or more per week | 0  1  2  3  4  5 |
| **F18. Lettuce (1 serving)** |  |
| O Never  O Less than 1 per month  O 1-3 times per month  O Once per week O 2-4 times per week O 5-6 times per week O Once per day O 2 or more times per day | 0  1  2  3  4  5  6  7 |
| **F19. Celery, cucumber (1 serving)** |  |
| O Never  O Less than 1 per month  O 1-3 per month  O Once per week O 2-4 per week O 5 or more per week | 0  1  2  3  4  5 |
| **F20. Avocado (1 serving)** |  |
| O Never  O Less than 1 per month  O 1-3 per month  O Once per week O 2-4 per week O 5 or more per week | 0  1  2  3  4  5 |
| **F21. Onion, spring onion, leek (1 serving)** |  |
| O Never  O Less than 1 per month  O 1-3 per month  O Once per week O 2-4 per week O 5 or more per week | 0  1  2  3  4  5 |
| **F22. Soybeans, tofu (1 serving)** |  |
| O Never  O Less than 1 per month  O 1-3 per month  O Once per week O 2-4 per week O 5 or more per week | 0  1  2  3  4  5 |
| **F23. Baked beans (1 serving)** |  |
| O Never  O Less than 1 per month  O 1-3 per month  O Once per week O 2-4 per week O 5 or more per week | 0  1  2  3  4  5 |
| **F24. Other beans, lentils e.g. chick peas, split peas (1 serving)** |  |
| O Never  O Less than 1 per month  O 1-3 per month  O Once per week O 2-4 per week O 5 or more per week | 0  1  2  3  4  5 |
| **Fruit** |  |
| **C. How many pieces of fruit do you eat (include all types)** |  |
| O None O Less than 1 per week  O 1-2 per week O 3-4 per week  O 5-6 per week  O Once per day O 2-3 per day O 4 or more per day | 0  1  2  3  4  5  6  7 |
| **F25. Canned fruit eg. peaches, Two fruits (1 serving)** |  |
| O Never  O Less than 1 per month  O 1-3 per month  O Once per week O 2-4 per week O 5 or more per week | 0  1  2  3  4  5 |
| **F26. Fruit salad (1 serving)** |  |
| O Never  O Less than 1 per month  O 1-3 per month  O Once per week O 2-4 per week O 5 or more per week | 0  1  2  3  4  5 |
| **F27. Dried fruit eg. sultanas, dried apricots (1 serving)** |  |
| O Never  O Less than 1 per month  O 1-3 per month  O Once per week O 2-4 per week O 5 or more per week | 0  1  2  3  4  5 |
| **F28. Apple or pear (1 serving)** |  |
| O Never  O Less than 1 per month  O 1-3 times per month  O Once per week O 2-4 times per week O 5-6 times per week O Once per day O 2 or more times per day | 0  1  2  3  4  5  6  7 |
| **F29. Orange, mandarin, grapefruit (1 serving)** |  |
| O Never  O Less than 1 per month  O 1-3 times per month  O Once per week O 2-4 times per week O 5-6 times per week O Once per day O 2 or more times per day | 0  1  2  3  4  5  6  7 |
| **F30. Banana (1 serving)** |  |
| O Never  O Less than 1 per month  O 1-3 times per month  O Once per week O 2-4 times per week O 5-6 times per week O Once per day O 2 or more times per day | 0  1  2  3  4  5  6  7 |
| **FS1. Peach, nectarine, plum or apricot (1 serving)** |  |
| O Never  O Less than 1 per month  O 1-3 per month  O Once per week O 2-4 per week O 5 or more per week | 0  1  2  3  4  5 |
| **FS2. Mango or paw-paw (1 serving)** |  |
| O Never  O Less than 1 per month  O 1-3 per month  O Once per week O 2-4 per week O 5 or more per week | 0  1  2  3  4  5 |
| **FS3. Pineapple (1 serving)** |  |
| O Never  O Less than 1 per month  O 1-3 per month  O Once per week O 2-4 per week O 5 or more per week | 0  1  2  3  4  5 |
| **FS4. Grapes, strawberries, blueberries (1 serving)** |  |
| O Never  O Less than 1 per month  O 1-3 per month  O Once per week O 2-4 per week O 5 or more per week | 0  1  2  3  4  5 |
| **FS5. Melon eg. watermelon, rockmelon, honeydew melon (1 serving)** |  |
| O Never  O Less than 1 per month  O 1-3 per month  O Once per week O 2-4 per week O 5 or more per week | 0  1  2  3  4  5 |
| **Total Score** | **190** |
