# Supplementary material for: Is Skin Coloration Measured by Reflectance Spectroscopy Related to Intake of Nutrient-Dense Foods? A Cross-Sectional Evaluation in Australian Young Adults
Source: Nutrients. 2017 Dec 23;10(1):11. doi: 10.3390/nu10010011 (PMC5793239; doi:10.3390/nu10010011)
Supplement: Supplementary file 1 [file nutrients-10-00011-s001.zip › Supplementary Table 1_ARFS.docx]

| **Food Group** | **Items giving 1 point** | **Items giving more than 1 point** | **ARFS** |
| --- | --- | --- | --- |
| *Vegetables* | 3-4 nightly meals with vegetables; ≥1per week of each of the following vegetables: potato, pumpkin, sweet potato, cauliflower, green beans, spinach, cabbage or Brussels sprouts, peas, broccoli, carrots, zucchini or eggplant or squash, capsicum, corn, mushrooms, tomatoes, lettuce, celery or cucumber, avocado, onion or leek or shallots/spring onion. | 2 points for ≥5 nightly meals  with vegetables | 21 |
| *Fruit* | ≥1 piece of fruit per day, ≥1 per week of each of the following fruit: canned fruit, fruit salad, dried fruit, apple or pear, orange or mandarin or grapefruit, banana, peach or nectarine or plum or apricot, mango or paw-paw, pineapple, grapes or strawberries or blueberries, melon (any variety). |  | 12 |
| *Protein foods- Meat/flesh* | ≤1 serve of mince meat per month but greater than never; 1 – 4 serve per week of: beef or lamb with or without sauce and/or vegetables per week chicken without batter or crumbing but with or without sauce and/or vegetables, pork with or without sauce and/or vegetables; ≥1 per week of fresh fish, canned tuna or salmon or sardines, other seafood (e.g. prawns, lobster). |  | 7 |
| *Vegetarian sources of protein* | ≥1 per week of the following: nuts (e.g. peanuts, almonds), nut butters, eggs, soybeans or tofu, baked beans, other beans or lentils (e.g. chickpeas, split peas). |  | 6 |
| *Breads and cereals (Grains)* | Usual bread choice is ‘other’ (e.g. rye, high-fiber white); ≥1 per week of the following: muesli, cooked porridge, breakfast cereal (e.g. Weet-bix, Nutri-grain, Cornflakes), bread or pita bread or toast, English muffin or bagel or crumpet, rice, other grains (e.g. couscous, burghul), noodles (e.g. egg noodles, rice noodles), pasta, tacos or burritos or enchiladas, clear soup with rice or noodles. | 2 points if usual bread choice is  ‘brown’ (multigrain or whole meal). | 13 |
| *Dairy* | ≥2 serves of: milk, yoghurt or cheese per day; ≥1 serve per week but ≤1 serves per day of flavored milk, ice cream, frozen yoghurt; ≥1 serve per week but ≤4 serves per day of cheese, cheese spread or cream cheese; ≥1 serve per week of plain milk, yoghurt (not frozen), cottage cheese or ricotta. | 2 points if usual type of milk is  reduced fat milk or skim milk,  or soy milk | 11 |
| *Water* | ≥4 glasses of water (including tap, unflavored bottled water, and unflavored mineral water). |  | 1 |
| *Spreads/ sauces* | ≥1 serve per week of: yeast extract spread; tomato or barbecue sauce |  | 2 |
| ***Total*** |  |  | **73** |

**Supplementary Table 1:** Scoring method for items in the ARFS
